# Clinical Lecture

**Published:** 1889-06

**Authors:** James Garretson


### CLINICAL LECTURE.

BY JAMES GARRETSON, A. M., M. D.

ESPECIALLY REPORTED FOR THE INTERNATIONAL.

GENTLEMEN :—Last week I brought before you this little child with the intention of performing on it the operation for fissure of the soft palate, but on finding that the child was somewhat unwell I sent it back into the wards till to-day. In the performance of this operation we need all possible favorable circumstances, and indeed even then I never approach it without trepidation, and doubt as to whether I do wisely. In fact the successes are so few that I am inclined to think it best in every case to use an obturator. After a little instruction and with an ordinary amount of skill any one can make an instrument of this character. Of course every dentist knows how to make an obturator; but this same knowledge may sometimes prove of great value to the physician. With some properly prepared wax first take an impression of the roof of the mouth. Perhaps you will say that I am old fogyish to make use of wax for such a purpose in this advanced age; but when for many years I have found a certain thing give satisfaction I am loath to give it up. Use plaster, if you choose, but as for me I shall continue with the wax. After having taken the impression, there will be a nodule on the wax corresponding to the fissure in the palate. This nodule can either be removed now, or after the plaster cast is taken, the corresponding depression in the cast can be filled with wax and smoothed with the finger. But this case we intend to treat surgically, and as the little patient is well under the influence of ether, I now proceed to freshen the surfaces we desire to oppose by cutting a small section from either side of the V-shaped fissure. This performance is easy enough, and though stitching the freshened edges together in the back of a baby's mouth is by no means so easy, it can be done, as you have just seen. But this is not the trouble. After having finished the operation, the surgeon may be pleased and inclined to congratulate himself on his success. The following day he will still be pleased, and so the next, and yet the the next. But on the day after that, upon looking into the mouth, he will probably notice that every one of those stitches placed with so much care is beginning to slough out, and will continue to slough in spite of the utmost efforts, until the parts are separated as widely as they were in the first place. I remember that some years ago I was surprised frequently to read in the English journals the remarkably good results in the cleft palate operations performed by Mr. Ferguson. As one of my assistants chanced to be going to England at that time, I asked him to make careful inquiries into the matter, and discover why our results compared so poorly. He did so, spending some months at Mr. Ferguson's clinics, and found that the percentage of real successes was not a whit more than our own; but that the accounts had mainly been published soon after the operation, before the final result could be known, and while everything was yet going smoothly.

### LEUCOPLAKIA BUCCALIS.

Here is a case of leucoplakia buccalis in a man of some forty-seven years of age, whom I have had before you several times within the last year.

This is a peculiar disease; you might almost say an anomalous one, and one that occupies, I believe, the borderland between malignant and non-malignant growths. Some call it ichthyosis of the mouth, some psoriasis, some eczema; others, other names. This diversity of naming is chiefly because the malady is so obscure, and in different instances simulates the appearances of those different affections. I call it leucoplakia buccalis, because its chief manifestation is that of white plaques or patches on the inner buccal surface. In the course of my specialty as an oral surgeon I have seen more cases of this trouble than fall to the lot of ordinary surgeons, and I have learned by experience, by sad experience, the folly and the harm of using radical measures; of trying to burn out the affected part with caustics. It is like striking with a wand a sleeping, hungry tiger. The hitherto quiescent and sluggish spot takes upon itself a swift and terrible growth. The temptation is now to use more caustics, and the effect is simply to spur on the disease, now truly malignant, to increased efforts at sapping the patient's life. This is a malady which beautifully illustrates our maxim of "when you know not what to do, do nothing."

There are few people, though, who having a certain trouble will submit to a course of doing nothing; but if I had this case of leucoplakia buccalis in my own mouth, I should follow the same treatment which I have advised this man to pursue—simply to use a wash of subnitrate of bismuth.

By acting on this advice the patient may live as long as any of us, and finally come to his end from some other cause; but if he allows the parts to be tormented by caustics, the course of his life will be suddenly and sadly changed.

I will now look into his mouth to see what condition we have at present. You may think I ought almost be ashamed, after dilating on the dangerous character of the disease, when I say that it looks almost well. Still I see a white line, a faint white line, extending from opposite the third lower molar to the malar process. Probably you would call it most insignificant; but it is the white line. Some may wonder why I do not cut it out. I have tried that, but it was before I fully comprehended the condition and when I was without any guide from the practice of others. I cut away, and cut away, and it would have been better for the patient had I been a thousand miles distant.

So I shall counsel this patient to continue the same treatment, reporting to us from time to time; and I advise you, when you see the white line, whatever you may call it, ichthyosis, leucoplakia, psoriasis, or eczema, let it alone.

### OSTEO-ODONTOMA.

Our last case is one of most peculiar interest; for it is not only wholly unique in my own experience, but I know of only one similar case that has been reported in all literature. That case is given fully in my system of Oral Surgery, as an abstract from a report by M. Fourget to the Royal Academy, together with a prize essay thereon. The two cases are so surprisingly similar that, if you will read the account referred to, you will have the characteristics of this. The patient is a young man of twenty-one, and according to his own account his history is as follows: With regard to dentition, he remembers nothing about his milk teeth; but as to the permanent set, he is certain that the two left lower bicuspids never made an appearance. The left side of the inferior maxilla has always been fully three times as large as the other side, showing that the tumor was congenital; and the enlargement grew in about the same relation to the right, giving him no particular inconvenience till a year and a half ago. At that time there appeared a sinus at the base of the left first molar from which pus continually exuded, and in addition there was swelling and other marked inflammatory symptoms about the jaw. Under the belief that the pus came from an abscess in the first molar, the patient's dentist tried to pull the tooth, but succeeded only in breaking it off. Pus continued to discharge and increase, till finally he went to a hospital in Boston, where they made an incision under the edge of the jaw, extending some six inches to the left of the median line, and removed, he says, a piece of bone as large as an English walnut. Before the operation, which was performed, October, 1888, he could separate his teeth not more than one-eighth of an inch on account of the great swelling in the submaxillary region. This part of the trouble was remedied by the operation, but the main tumor still was there, and pus exuded as before.

His dentist then advised him to consult with me.

Upon first making an examination, I thought I had to deal simply with a case of necrosis of the lower jaw; for on the left side could be seen a large and apparently spongy mass, from which oozed an offensive discharge. But the probe soon disclosed the fact that we had something entirely different and more formidable to treat. On all sides a solid bony mass was felt, and the sharp burr of the powerful surgical engine found a surface as hard and as smooth as a billiard ball. The former way of treating a case like this would have been to make an incision along the under border of the lower jaw, dissect up the flap, and remove the entire half of the jaw. This is a quick and easy method, but our surgical engine will remove the tumor and yet leave the jaw there for both service and looks. We have operated on five different occasions, because the patient will take no ether, and because of the slowness of our progress; but we expect to finish before you to-day. As the patient lay on the table at first, the size of the tumor could easily be seen. It reached down to a level with the upper border of the thyroid cartilage, was about three inches in diameter on its short axis, and extended from the median line to half way up the ramus of the jaw. Laterally considered, the left side of the face is about an inch and a half wider than the right. The pus would seem to have been developed by the effort of nature to throw off an offending foreign body, and as for the composition of the tumor, it consists of a heterogeneous mass of bone, and of dentinal tissues, containing in its substance at least one partly formed tooth, the deciduous lateral incisor, and altogether having a formation strikingly similar to the singular case related by M. Fourget.

